# Nurses’ knowledge on phlebotomy in tertiary hospitals in China: a cross-sectional multicentric survey

**DOI:** 10.11613/BM.2018.010703

**Published:** 2017-11-24

**Authors:** Qian Cai, Yunxian Zhou, Dangan Yang

**Affiliations:** 1 School of Nursing, Zhejiang Chinese Medical University, Hangzhou, China; 2 Department of Laboratory Medicine, The First Affiliated Hospital, College of Medicine, Zhejiang University, Hangzhou, China

**Keywords:** pre-analytical phase, venous blood sampling, knowledge, nurses, phlebotomy

## Abstract

**Introduction:**

In China, phlebotomy practice is mostly executed by nurses instead of phlebotomists. Our hypothesis was that these nurses may lack of knowledge on phlebotomy, especially factors influencing quality of blood samples. This study aims to assess the overall nurses’ knowledge on phlebotomy to provide reference for improving blood sampling practice in China.

**Materials and methods:**

A survey was conducted involving nurses from 4 regions and 13 hospitals in China. A phlebotomy knowledge questionnaire was designed based on the Clinical and Laboratory Standards Institute H3-A6 guidelines, combining with the situations in China. Descriptive analysis and binary logistic regression analysis were used to analyze the knowledge level and its influencing factors.

**Results:**

A total of 3400 questionnaires were distributed and 3077 valid questionnaires were returned, with an effective return rate of 90.5%. The correct rates of patient identification, hand sanitization, patient assessment, tube mixing time, needle disposing location and tube labelling were greater than 90%. However, the correct rates of order of draw (15.5%), definition of an inversion (22.5%), time to release tourniquet (18.5%) and time to change tube (28.5%) were relatively low. Binary logistic regression analysis showed that the correct rates of the aforementioned four questions were mainly related to the regional distribution of the hospitals (P < 0.001).

**Conclusions:**

The knowledge level on phlebotomy among Chinese nurses was found unsatisfactory in some areas. An education program on phlebotomy should be developed for Chinese nurses to improve the consistency among different regions and to enhance nurse’s knowledge level on phlebotomy.

## Introduction

Venous blood sampling is an important procedure affecting laboratory results. It consists of several discrete steps, including patient preparation, collection and handling of blood specimens, and each step is prone to errors. Since laboratory results are the basis for 60 - 80% of medical decisions, any error in the phlebotomy process could cause serious consequences (*1*). Studies have reported that up to 65% of laboratory errors occurred in the pre-analytical phase, and this phase has been proved difficult to standardize (*2*).

The quality of venous blood specimen collected in the pre-analytical phase has a close relationship with knowledge on phlebotomy (*3*). Thus, evaluating the phlebotomy knowledge could be the first step in reducing specimen rejection and improving specimen quality. Some European and African studies have shown that healthcare professionals had an inadequate knowledge or imperfect practices of phlebotomy (*4*-*6*). The correct rates of patient identification, wearing gloves, tourniquet application time, order of draw, mixing of tubes were found to be unacceptably low (*4*-*6*). A questionnaire survey in Turkey revealed that nurses’ awareness of blood sample collection procedures needed to be improved (*7*).

In some European countries (the Netherlands, Belgium, Ireland and the United Kingdom) and the United States, venous blood samples are collected by phlebotomist, who have received professional phlebotomy education and practice training (*3*, *8*). Moreover, a well-documented instruction for phlebotomy is usually available during sampling process in these countries. The Clinical and Laboratory Standards Institute (CLSI) H3-A6 Procedures for the Collection of Diagnostic Blood Specimens by Venipuncture; Approved Standard-Sixth Edition (CLSI H3-A6 guidelines) and World Health Organization (WHO) Guidelines on Drawing Blood: Best Practices in Phlebotomy were two widely used phlebotomy guidelines in European countries (*1*, *3*, *9*, *10*).

In China, blood specimen collection is mainly performed by nurses. There is little systematic training on blood specimen quality control in Chinese nursing education (*11*). In addition, there are no national guidelines in China and the phlebotomy regulations vary widely among different hospitals (*12*). Since laboratories in China have little collaboration with nursing departments, nurses received inadequate guidance from laboratory staff on improving blood specimen quality (*11*). Limited literature showed that one of the biggest issues resulting in low-quality blood specimen in China was nurses’ inadequate knowledge on blood specimen collection, particularly regarding patient preparation and the sampling phase of blood specimen collection (*13*). Therefore, our hypothesis was that Chinese nurses may lack of knowledge on phlebotomy, especially factors influencing quality of blood samples. The survey was carried out to assess the knowledge on venous blood sampling among Chinese clinical nurses in tertiary hospitals, to provide references for improving blood sampling practice in China.

## Materials and methods

### Study design

A multistage sampling was used in this study. In the first stage, a stratified sampling was used to select 13 Grade A tertiary hospitals (representing the highest level of hospitals in China) from 4 economic regions of China. Specialized hospitals and the hospitals unwilling to participate were excluded. After that, at least four departments (including at least one medical ward and one surgical ward) from each of the 13 hospitals were selected. Finally, a convenience sampling (a specific type of non-probability sampling method) was used to invite all registered nurses on duty in those selected departments on the investigation day to participate. Nurses were excluded if they held administration positions without direct patient care responsibilities.

A cross-sectional multicentric survey was conducted from November to December 2015 by four research nurses. They were trained together before data collection. The training included the research goals, sampling methods, correct delivery and returning process for the questionnaires. The questionnaires were delivered face to face, and the nurses completed the questionnaires anonymously and returned them to the investigators on the same day. No incentives were offered for participation. This study was approved by the ethics committee of the First Affiliated Hospital, College of Medicine, Zhejiang University, China. Informed consent was obtained from each investigated nurse.

### Methods

Based on the CLSI H3-A6 guidelines, combining with the situation in China, a draft questionnaire on knowledge of venous blood sampling was designed (*9*). Three nursing experts and two clinical laboratorians who specialize in specimen collection and management were invited to evaluate the content and expression of the questionnaire and revisions were made accordingly. The final version of the questionnaire comprised of two sections: demographic profiles of the nurses, such as age, education, hospital, department, professional title and questions (including single-choice questions, multiple-choice questions, and fill-in the blanks) on knowledge of venous blood sample collection. The latter part consists of issues from pre-sampling phase, sampling phase and post-sampling phase. A missing rate of no greater than 20% in both sections was considered as a valid questionnaire.

### Statistical analysis

Frequency, percentage, quartile range, and median were used to describe nurses’ characteristics and knowledge level of phlebotomy. The Kolmogorov-Smirnov test was used to assess the normality of distribution of numerical data. Binary logistic regression analysis was conducted to determine the influence factors for the right or wrong mastery on the questions of order of draw, definition of an inversion, time to release tourniquet and time to change tube, using age, education, hospital region, department and professional title as independent variables, and the P-value was calculated by the Wald Chi square test. A P-value less than 0.05 was considered statistically significant. SPSS 19.0 (SPSS Inc., Chicago, IL, USA) was used for statistical analysis.

## Results

A total of 3400 questionnaires were distributed across 13 hospitals from 4 regions of China (2 hospitals from the northeast region, 3 hospitals from the central region, 4 hospitals from the east region, and 4 hospitals from the west region), and 3077 valid questionnaires were returned, with an effective return rate of 90.5%. The demographic profile of the investigated nurses was shown in Table 1.

**Table 1:** The demographic profiles of the investigated nurses in the survey

**Variables**	**Response rate,** **N (%)**	**Missing rate,** **N (%)**
**Age (years)**		
≤ 25	744 (24.2)	116 (3.8)
26 - 35	1812 (58.9)	
36 - 45	322 (10.4)	
≥ 46	83 (2.7)	
**Education level**		
Associate degree or less	1092 (35.5)	67 (2.2)
Bachelor degree or above	1918 (62.3)	
**Professional title**		
Staff nurses	1045 (34.0)	119 (3.9)
Senior nurses	1418 (46.1)	
Chief nurses or above	495 (16.1)	
**Department**		
Medical ward	1427 (46.4)	89 (2.9)
Surgical ward	990 (32.2)	
Obstetrics and gynecological ward	165 (5.4)	
Outpatient and Emergency department	280 (9.1)	
Other wards	126 (4.1)	

Almost all the investigated nurses were aware that patient identification should be confirmed prior to venous blood sampling. However, only 69.7% of the nurses knew what identifiers should be used for identifying a patient and 58.8% of the nurses knew the right procedures to identify a patient who is conscious. In addition, the correct rate of hand sanitization and patient assessment were no less than 95%. The detailed results on phlebotomy knowledge of the pre-sampling phase were shown in Table 2.

**Table 2:** Knowledge of pre-sampling phase of the investigated nurses in the survey

**Questions**	**Response rate,** **N (%)**	**Missing rate,** **N (%)**	**Correct rate,** **N (%)^‡^**
**Should patient identity be confirmed prior to venous blood sampling?**			
Yes*	3060 (99.4)*	17 (0.6)	3060 (99.4)
No	0 (0)		
**What identifiers should be used when identifying a patient?** (multi-choice)			
Patient’s full name^†^	3019 (98.1)^†^	31 (1.0)	2144 (69.7)^§^
Patient’s age	2068 (67.2)		
Patient’s bed number	2754 (89.5)		
Patient’s identification number^†^	2153 (70.0)^†^		
**What are the right procedures to identify a patient who is conscious?** (multi-choice)			
Ask a patient to give his/her full name*	2841 (92.3)*	68 (2.2)	1810 (58.8)^§^
Nurse state a patient’s full name or bed number	1614 (52.5)		
Check a patient’s bracelet*	1896 (61.6)*		
Check a patient’s bed tag	2238 (72.7)		
**How many times should a test request form be verified?**			
1	23 (0.7)	1236 (40.2)	1544 (50.2)
2	177 (5.7)		
3^†^	1544 (50.2)^†^		
≥ 4	97 (3.2)		
**What are the right approaches for hand sanitization?** (multi-choice)			
Hand disinfectant^†^	2836 (92.2)^†^	75 (2.4)	2999 (97.5)^||^
Soap and water^†^	93 (3.0)^†^		
Disinfectant gel^†^	70 (2.3)^†^		
Not necessary because gloves were used	3 (0.1)		
**What should be inspected about the supplies?** (multi-choice)			
Intact phlebotomy device package*	2849 (92.5)*	26 (0.8)	2411 (78.4)^§^
Applicable expiration dates of phlebotomy devices*	2797 (90.9)*		
Looseness or defects of the tube closure*	2848 (92.5)*		
Appropriate tubes according to the test requests*	2632 (85.5)*		
**What patient assessment should be included before venous blood sampling?** (multi-choice)			
Medication use^†^	1845 (60.0)^†^	19 (0.6)	1106 (95.0)
Diet restriction^†^	2922 (95.0)^†^		
Strenuous exercise^†^	1433 (46.6)^†^		
History of needle syncope^†^	1829 (59.4)^†^		
Others (*i.e.* health history or allergy history) ______^†^	81 (2.6)		
*Correct options based on the CLSI H3-A6 guidelines. ^†^Correct options based on the situation in China. ^‡^A column of correct rate was established because some questions were multiple choice questions in the Table 2. ^§^The question was considered correct only if all the correct options were chosen. ^||^The question was considered correct if at least one of the correct options was chosen. ^¶^The question was considered correct as long as the answer of diet restriction was chosen.			

As Table 3 showed, the correct rates on the preferred vein for venipuncture, diameter of disinfection, proper time and approach for mixing tubes were relatively high (more than 85%). Although 78.1% of the nurses knew proper tourniquet application time, the correct rates on the tourniquet releasing time (18.5%) and tourniquet applying location (29.6%) were relatively low. The correct rate of tourniquet releasing time was associated with the regions of hospitals (P < 0.001), compared to the east region, the central region had the highest correct rate (odds ratio, OR = 0.68, 95% CI 0.51 - 0.92), followed by northeast region (OR = 0.69, 95% CI 0.53 - 0.91), while the west region had the lowest correct rate (OR = 1.32, 95% CI 1.02 - 1.71) (Table 4). In addition, around two thirds of the investigated nurses knew that gloves should be worn during phlebotomy, yet only about one third knew the proper time to put on gloves. Furthermore, only 15.5% of the nurses knew the right order of draw during multi-tube sampling (Table 5). Its correct rate was associated with the regions of hospitals (P < 0.001), compared to the east region, the northeast region had the highest correct rate (OR = 0.07, 95% CI 0.05 - 0.10), followed by the west region (OR = 0.36, 95% CI 0.26 - 0.49), while the central region had the lowest correct rate (OR = 2.17, 95% CI 1.11 - 4.26) (Table 4). Regarding to the proper time to change tube during multi-tube sampling, the correct rate was 28.5%. Its correct rate was associated with the regions of hospitals (P < 0.001) and age of the nurses (P < 0.001), compared to the east region, the northeast region had a higher correct rate (OR = 0.24, 95% CI 0.19 - 0.31); and the older the nurses, the higher the correct rate (OR = 0.96, 95% CI 0.94 - 0.99) (Table 4). As to the definition of an inversion, only 22.5% of the nurses knew the correct answer (Table 3). Its correct rate was associated with regions of hospitals (P < 0.001) and departments of the nurses (P = 0.001), compared to the east region, the central region had the highest correct rate (OR = 0.49, 95% CI 0.37-0.65), followed by the northeast region (OR = 0.52, 95% CI 0.40 - 0.68) and the west region (OR = 0.67, 95% CI 0.54 - 0.85), while outpatient and emergency department (OR = 0.61, 95% CI 0.46 - 0.82) had a higher correct rate compared to inpatient department (Table 4). The knowledge of mix by inverting times for blood collection tubes was shown in Table 6. Among all the investigated nurses, 1012 failed to respond to this question, with a missing rate of 32.9%. As Table 6 showed, in four out of six tubes, the medians of responded times of tube inversions were lower than those of recommended times.

**Table 3:** Knowledge of sampling phase of the investigated nurses in the survey

**Questions**	**Response rate,** **N (%)**	**Missing rate,** **N (%)**	
**Which vein is preferred for venipuncture?**			
Median cubital vein / median vein*	2683 (87.2)*	62 (2.0)	
Basilic vein	289 (9.4)		
Other veins	43 (1.4)		
**Where is the proper location to apply a tourniquet?**			
3.5 - 5.0 cm above the venipuncture site	336 (10.9)	78 (2.5)	
> 5.0 - < 7.5 cm above the venipuncture site	1577 (51.2)		
7.5 - 10.0 cm above the venipuncture sit^e^*	910 (29.6)*		
> 10.0 - < 12.5 cm above the venipuncture site	159 (5.2)		
12.5 - 15.0 cm above the venipuncture site	17 (0.6)		
**How long can tourniquet application last?**			
≤ 60 seconds*	2402 (78.1)*	629 (20.4)	
> 60 seconds	46 (1.5)		
**Should gloves be worn during phlebotomy?**			
Yes*	2021 (65.7)*	84 (2.7)	
No	972 (31.6)		
**When is the proper time to put on gloves?**			
Before assembling supplies	299 (9.7)	683 (22.2)	
Before performing venipuncture*	2062 (67.0)*		
Others______	33 (1.1)		
**What is the proper diameter of disinfection?**			
< 5.0 cm	48 (1.5)	356 (11.6)	
≥ 5.0 cm^†^	2673 (86.9)^†^		
**What is the proper angle of needle insertion?**			
≤ 30°*	1896 (61.6)*	124 (4.0)	
> 30 - ≤ 45°	1048 (34.1)		
> 45°	9 (0.3)		
**When is the proper time to release the tourniquet?**			
After succeeding in performing venipuncture	898 (29.2)	117 (3.8)	
As soon as blood flow is established*	569 (18.5)*		
When blood half-fills the first tube	143 (4.6)		
When blood fills the first tube	200 (6.5)		
When blood fills the last tube	1150 (37.4)		
**When is the proper time to change tube during multi-tube sampling?**			
Until the vacuum is exhausted and blood flow ceases*	877 (28.5)*	179 (5.8)	
Until the volume of the blood sample is sufficient based on your judgement	2021 (65.7)		
**When is the proper time to mix the blood collection tube that contains additives?**			
Mix each tube instantly after blood sampling*	2889 (93.9)*	64 (2.1)	
Mix all tubes together after blood sampling is completed	124 (4.0)		
**What is the proper approach for mixing blood with additives?**			
Inverting up and down*	2666 (86.6)*	91 (3.0)	
Vibrating left and right	320 (10.4)		
**How many times of mixture do you consider it is if a tube is fully inverted for 180° and then returned to the** **initial position?**			
Once*	691 (22.5)*	174 (5.6)	
Twice	2212 (71.9)		
**What is the proper approach for removing the needle and applying pressure?**			
Withdraw the needle prior to applying pressure*	2061 (67.0)*	54 (1.7)	
Withdraw the needle after applying pressure	962 (31.3)		
*Correct options based on the CLSI H3-A6 guidelines. ^†^Correct options based on the situation in China.			

**Table 4:** Logistic regression analysis of the mastery of order of draw, definition of one inversion, time to release tourniquet and time to change tubes

**Variables**	**Categorical Variables**	**β**	**SE**	**Wals**	**OR (95% Cl)**	**P**
**Order of draw**	**Age (years)**	0.024	0.015	2.504	1.02 (0.99 - 1.06)	0.114
	**Education level**					
	Associate degree or less*	-0.026	0.132	0.039	0.97 (0.75 - 1.26)	0.843
	Bachelor degree or above					
	**Professional title**			8.950		**0.011**
	Staff nurses*					
	Senior nurses	0.169	0.154	1.199	1.18 (0.88 - 1.60)	0.274
	Chief nurses or above	- 0.429	0.272	2.497	0.65 (0.38 - 1.11)	0.114
	**Department**					
	Hospital wards*	0.000	0.195	0.000	1.00 (0.68 - 1.47)	1.000
	Outpatient & Emergency department					
	**Hospital region**			322.364		**< 0.001**
	East region*					
	Northeast region	- 2.617	0.159	270.396	0.07 (0.05 - 0.10)	**< 0.001**
	Central region	0.775	0.344	5.089	2.17 (1.11 - 4.26)	**0.024**
	West region	- 1.026	0.157	42.812	0.36 (0.26 - 0.49)	**< 0.001**
**Definition of one inversion**	**Age (years)**	- 0.012	0.012	1.040	0.99 (0.97 - 1.01)	0.308
	**Education level**					
	Associate degree or less*	- 0.206	0.108	3.659	0.81 (0.66 - 1.01)	0.056
	Bachelor degree or above					
**Definition of one inversion**	**Professional title**			2.819		0.244
	Staff nurses*					
	Senior nurses	0.206	0.123	2.786	1.23 (0.97 - 1.56)	0.095
	Chief nurses or above	0.254	0.218	1.363	1.29 (0.84 - 1.98)	0.243
	**Department**					
	Hospital wards*	- 0.489	0.145	11.316	0.61 (0.46 - 0.82)	**0.001**
	Outpatient & Emergency department					
	**Hospital region**			38.147		**< 0.001**
	East region*					
	Northeast region	- 0.651	0.133	24.064	0.52 (0.40 - 0.68)	**< 0.001**
	Central region	- 0.714	0.143	24.972	0.49 (0.37 - 0.65)	**< 0.001**
	West region	- 0.394	0.118	11.099	0.67 (0.54 - 0.85)	**0.001**
**Time to release tourniquet**	**Age (years)**	- 0.007	0.012	0.287	0.99 (0.97 - 1.02)	0.592
	**Education level**					
	Associate degree or less*	0.153	0.115	1.781	1.17 (0.93 - 1.46)	0.182
	Bachelor degree or above					
	**Professional title**			2.189		0.335
	Staff nurses*					
	Senior nurses	- 0.061	0.135	0.205	0.94 (0.72 - 1.22)	0.651
	Chief nurses or above	- 0.314	0.229	1.888	0.73 (0.47 - 1.14)	0.169
	**Department**					
	Hospital wards*	- 0.212	0.159	1.767	0.81 (0.59 - 1.11)	0.184
	Outpatient & Emergency department					
	**Hospital region**			23.968		**< 0.001**
	East region*					
	Northeast region	- 0.367	0.139	6.965	0.69 (0.53 - 0.91)	**0.008**
	Central region	- 0.382	0.152	6.317	0.68 (0.51 - 0.92)	**0.012**
	West region	0.279	0.132	4.496	1.32 (1.02 - 1.71)	**0.034**
**Time to change tubes**	**Age (years)**	- 0.042	0.011	13.493	0.96 (0.94 - 0.98)	**< 0.001**
	**Education level**					
	Associate degree or less*	0.085	0.104	0.673	1.09 (0.89 - 1.34)	0.412
	Bachelor degree or above					
	**Professional title**			5.431		0.066
	Staff nurses*					
	Senior nurses	- 0.246	0.123	3.994	0.93 (0.61 - 1.40)	**0.046**
	Chief nurses or above	- 0.078	0.211	0.136	0.78 (0.61 - 1.00)	0.712
	**Department**					
	Hospital wards*	0.072	0.153	0.222	1.08 (0.80 - 1.45)	0.638
	Outpatient & Emergency department					
	**Hospital region**			157.368		**< 0.001**
	East region*					
	Northeast region	- 1.416	0.124	129.659	0.24 (0.19 - 0.31)	**< 0.001**
	Central region	0.133	0.155	0.742	1.14 (0.84 - 1.55)	0.389
	West region	- 0.013	0.113	0.013	0.99 (0.79 - 1.23)	0.909
*Control group. β - regression coefficient. SE - Standard Error. Wals - Wald Chi square. OR - Odds Ratio. 95% Cl - 95% confidence interval. P - P-value. P < 0.05 was considered statistically significant.						

**Table 5:** Knowledge on order of draw during multi-tube sampling

Fill the number 1 - 6 in blanks for correct order of draw during multi-tube sampling*	**Right response** **rate, N (%)**	**Wrong response rate, N (%)**	**Missing rate,** **N (%)**
___Red / Yellow closure (Serum tube with or without clot activator, with or without gel plasma separator)	476 (15.5)	2120 (68.9)	481 (15.6)
___Blood culture tube			
___Lavender closure (EDTA tube)			
___Green closure (Heparin tube)			
___Blue closure (1:9 Sodium citrate tube)			
___Gray closure (Glycolytic inhibitor tube)			
*The right answer is 3 - red / yellow closure, 1 - blood culture tube, 5 - lavender closure, 4 - green closure, 2 - blue closure, 6 - gray closure.			

**Table 6:** Knowledge of mix by inverting times for blood collection tubes

**Fill the number of inversions in the blank for the different tubes respectively**	**Recommended number of tube inversions**	**Responded number of tube inversions (Quartile)**			**Right response rate, N (%)**
**25th**	**50th**	**75th**	
Red closure (Non-additive serum tube)	0	0	2	5	285 (9.3)
Yellow closure (Tube with clot activator and gel plasma separator)	5	0	2	5	732 (23.8)
Lavender closure (EDTA tube)	8	2	4	8	856 (27.8)
Green closure (Heparin tube)	8	2	3	6	975 (31.7)
Blue closure (1:9 Sodium citrate tube)	3-4	2	4	5	1255 (40.8)
Grey closure (Glycolysis inhibitor tube)	8	2	3	6	676 (22.0)

The correct rates on where to dispose needle, post-venipuncture education and tube labelling were relatively high (more than 85%). However, half of the investigated nurses considered the disposal of the needle after detaching the device with both hands acceptable practice. Around one quarter of the nurses stated that there was no need of documenting the time and person of blood collection. The detailed results on the phlebotomy knowledge in the post-sampling phase were shown in Table 7.

**Table 7:** Knowledge of post-sampling phase of the investigated nurses in the survey

**Questions**	**Response rate, N (%)**	**Missing rate, N (%)**
**Where is the right location for applying pressure to stop the bleeding?**		
Over the venipuncture site*	1774 (57.7)*	366 (11.9)
Beneath the venipuncture site	90 (2.9)	
On the venipuncture site	823 (26.7)	
Uncertain	24 (0.8)	
**How long should pressure applying last for a patient with risk of bleeding?**		
2 minutes	135 (4.4)	422 (13.7)
3 minutes	483 (15.7)	
4 minutes	60 (1.9)	
≥ 5 minutes*	1977 (64.3)*	
**Should post-venipuncture education (*e.g.* pressure applying approach, location) be provided to patients or their family members?**		
Yes^†^	2701 (87.8)^†^	331 (10.7)
No	45 (1.5)	
**Where to dispose the needle after phlebotomy?**		
Medical waste dustbin	28 (0.9)	51 (1.6)
Sharps container*	2996 (97.4)*	
Common waste dustbin	2 (0.1)	
**What is the proper approach to dispose the needle?**		
Dispose directly with one hand^†^	1481 (48.1)^†^	57 (1.9)
Dispose after detaching the device with both hands	1539 (50.0)	
**When is the proper time to label blood collection tubes?**		
Label the tube when verifying the test request form before sampling^†^	2782 (90.4)^†^	53 (1.7)
Label the tube after filling, in presence of the patient	234 (7.6)	
Label the tube after leaving the patient	8 (0.3)	
**Should the person collecting specimen and the time of collection be recorded?**		
Yes*	2253 (73.2)*	63 (2.1)
No	761 (24.7)	
*Correct options based on the CLSI H3-A6 guidelines. ^†^Correct options based on the situation in China.		

## Discussion

To the best of our knowledge, few surveys were conducted on assessing the knowledge level of phlebotomy in Chinese nurses up to now. In addition, the surveys were conducted more than 5 years ago, and included only one or two hospitals, unable to provide a reliable reference for the overall situation of nurses’ knowledge on phlebotomy at present (*14*, *15*). The level of knowledge on phlebotomy among Chinese nurses was found to be not quite satisfactory. Compared with previous survey in China, some progress had been made in the steps of patient preparation, tourniquet application time and test tube labeling, while the survey also showed that the mastery of some knowledge on venous blood sampling needed to be improved (*14*, *15*).

A high accuracy of patient identification is crucial to ensure patient safety, and a proper patient identification must rely on at least two independent identifiers. In our survey, the identifiers selected for identifying a patient include bed number (89.5%), and the approaches for identifying include nurses stating a patient’s bed number. According to the CLSI H3-A6 guidelines and Joint Commission International requirements, bed number can’t be used as an independent identifier for patient identification, due to the fact that it is not a unique index representing a patient (*9*, *16*). A misidentification could occur if judging only via the bed number and potentially causes serious consequences. Therefore, it is important to select the full name and patient identification number as the identifying variables.

The CLSI H3-A6 guidelines recommend that tourniquet application time should not exceed one minute and tourniquet should be released as soon as possible after the blood begins to flow to prevent spurious variation of several plasma analytes. The correct rate on tourniquet releasing time was low (18.5%) in our survey. A European survey showed that 43.0% of the nurses did not release the tourniquet when the first test tube had blood inflows (*4*). It is possible that nurses would not release tourniquet before the blood collection process is completed to avoid vein collapsing. Further studies are needed to explore whether this concern is evidence based or not.

Nonsterile gloves should be worn during sampling phase to prevent the spread of blood-borne pathogens among health-care professionals. As our investigation showed, nearly one third of the nurses were not aware of this requirement. A Kenyan survey showed that more than two-thirds of the nurses did not use a new pair of gloves in phlebotomy, while a European survey also found that more than half of the nurses did not wear gloves correctly during blood collection (*4*, *5*). Mbah considered this issue could be caused by a limited supply of gloves or a concern that a vein would not be able to feel with gloves on (*17*). We thought it was also possible that the nurses did not attach adequate importance on wearing gloves during sampling. Therefore, it is important to promote a routine use of gloves during blood sample collection.

A correct order of draw prevents the sample being contaminated by additives from previous tubes, which could cause an erroneous result. In fact, contamination of blood sample occurred frequently if the correct order of test tubes was not strictly followed (*18*). As our investigation showed, only a small proportion of the nurses (15.5%) were aware of the correct order of multi-tube blood sampling whereas most of the nurses (91.9%) in Europe knew the answer (*4*). One cause of the low correct rate among Chinese nurses on order of draw was related to the fact that no unified standard on order of draw existed in China, while the order taught in different textbooks varied widely (*9*, *13*). In addition, some studies have pointed out that an incorrect order of draw would not cause contamination when samples were taken under ideal phlebotomy conditions (*19*). However, other authors have argued that clinical blood collection was rarely carried out in an ideal condition and therefore it was recommended that phlebotomy should be executed in the order described in the CLSI H3-A6 guidelines (*20*). Furthermore, there are many types of vacuum tubes used in phlebotomy, thus it is not easy to keep the right combination order of the tubes in mind. To promote compliance with the right order of draw, we recommend the tube manufacturer to label the correct order of draw on the test tubes. Alternatively, the laboratory information system can be improved by adding the correct order of draw into the bar code of information printing system as a reminder.

Proper mixing is important for homogenizing the collected blood with the additives in the test tubes (*12*). An inversion means the test tube is inverted up and down for 180 degrees. As the survey showed, few nurses (22.5%) in China were aware of the correct definition of an inversion and the mix by inverting times for most tubes was below the standard. Giving that the Chinese nurses had a misunderstanding on the definition of an inversion, the actual mixing time might be even lower. One study in Turkey showed that the correct rate of tube mixing after blood collection was 44.1%, while an observation study from Kenya also showed that nearly 90% of the sampling tubes were not properly mixed (*5*, *7*). Inadequate mixing of blood and anti-coagulations would increase the risk of blood coagulation. There was report showing that China had a high clot rate of rejected specimens for complete blood count testing (77.38%), which could possibly be caused by insufficient mixing (*21*). Therefore, it is necessary for nurses to master the correct mixing times of various types of test tubes. Since it is not easy for nurses to remember the correct number of inversions for different blood collection tubes, we recommend manufactures to label the mixing times on the tubes. Adding the mixing times on the bar code of the laboratory information system by hospital information department could be another option.

During blood collection, it is important to allow the tube to fill until the vacuum is exhausted and blood flow ceases. This will ensure there is a correct ratio of blood to additives for tubes that contain additives. In our survey, more than two-thirds of the nurses answered this question incorrectly, which might be related to the fact that nurses were used to judge the blood volume based on personal experience in the past when syringe and needle were commonly used. A Kenya study found that 87% of the volume of the sample was inadequate (*5*). Too high final concentration of the additive may result in pre-analytical errors such as haemolysed sample, changes in cell morphology, prolonged coagulation times (*22*). Thus, it is necessary to educate nurses about the importance of correct ratio of blood to additives.

Among the three phases of venous blood sample collection, the mastery of knowledge of the sampling phase was the most unsatisfactory. Compared with knowledge on the venous blood sampling procedure, Chinese nurses knew less on blood specimen quality control, probably because the nurses were more concerned about the phlebotomy procedure instead of quality of blood sample. This may also reflect the inadequacy of phlebotomy training received by Chinese nurses. This result is consistent with the previous findings in China (*12*, *14*).

The order of draw, definition of an inversion, time to release tourniquet, and time to change tube were the four questions with relative low correct rates, which were mainly influenced by the region of the hospitals as shown in the logistic regression analysis. We assume this might be related to the different policy and training on phlebotomy of the hospitals. It is urgently needed to establish a national guideline for blood collection to enhance the consistency among different hospitals and improve the quality of specimen collected.

There are some limitations in our study. First, the questionnaire for investigation was self-designed without undergoing a thorough reliability and validity test. In addition, some questions had a high missing rate, which might bias our results. It is recommended that a standardized questionnaire would be developed to investigate the mastery of blood sampling knowledge among Chinese nurses in future. Second, our main research aim is to assess nurses’ overall knowledge on phlebotomy in tertiary hospitals in China, and in future it is also worthwhile to explore factors influencing knowledge level. Third, considering there is a knowledge-behavior gap, we highly recommend that an observational study of the blood sampling practice among nurses would be conducted in future for further assessing the compliance of phlebotomy with the guidelines. Despite the aforementioned limitations, our survey had a relatively large sample size covering a wide range of regions in China, and the questions addressed in the questionnaire were quite comprehensive. Thus, we believed that this survey provided important baseline data for understanding the status on the mastery of phlebotomy knowledge among the nurses in China.

In conclusion, the mastery of phlebotomy knowledge among Chinese nurses still has much room to improve. Cooperation between the nursing department and the laboratory should be encouraged to develop targeted intervention for Chinese nurses to enhance the mastery of phlebotomy knowledge, particularly in the area of quality control of blood samples.
