# Corrigendum to: Nurses’ knowledge on phlebotomy in tertiary hospitals in China: a cross-sectional multicentric survey

**DOI:** 10.11613/BM.2018.011202

**Published:** 2018-01-10

**Authors:** Qian Cai, Yunxian Zhou, Dagan Yang

**Affiliations:** 1 School of Nursing, Zhejiang Chinese Medical University, Hangzhou, China; 2 Department of Laboratory Medicine, The First Affiliated Hospital, College of Medicine, Zhejiang University, Hangzhou, China

This is a correction of *Biochemia Medica* 2018;28(1):010703. DOI: https://doi.org/10.11613/BM.2018.010703

Since the publication of this article, the authors have noticed that the surname of the last author in the byline was published incorrectly. The correct byline is presented above. The authors apologize for any inconvenience caused to the readers.

